# Effect of prevaccination blood and T-cell phenotypes on antibody responses to a COVID-19 mRNA vaccine

**DOI:** 10.1093/intimm/dxaf013

**Published:** 2025-03-21

**Authors:** Yu Hidaka, Norihide Jo, Osamu Kikuchi, Masaru Fukahori, Takeshi Sawada, Yutaka Shimazu, Masaki Yamamoto, Kohei Kometani, Miki Nagao, Takako E Nakajima, Manabu Muto, Satoshi Morita, Yoko Hamazaki

**Affiliations:** Department of Biomedical Statistics and Bioinformatics, Graduate School of Medicine, Kyoto University, Kyoto, Japan; Department of Life Science Frontiers, Center for iPS Cell Research and Application (CiRA), Kyoto University, Kyoto, Japan; Alliance Laboratory for Advanced Medical Research, Graduate School of Medicine, Kyoto University, Kyoto, Japan; Department of Medical Oncology, Graduate School of Medicine, Kyoto University, Kyoto, Japan; Clinical Bio-Resource Center, Kyoto University Hospital, Kyoto, Japan; Center for Cancer Immunotherapy and Immunobiology, Kyoto University, Kyoto, Japan; Department of Early Clinical Development, Graduate School of Medicine, Kyoto University, Kyoto, Japan; Kyoto Innovation Center for Next Generation Clinical Trials and iPS Cell Therapy (Ki-CONNECT), Kyoto University Hospital, Kyoto, Japan; Department of Early Clinical Development, Graduate School of Medicine, Kyoto University, Kyoto, Japan; Kyoto Innovation Center for Next Generation Clinical Trials and iPS Cell Therapy (Ki-CONNECT), Kyoto University Hospital, Kyoto, Japan; Department of Early Clinical Development, Graduate School of Medicine, Kyoto University, Kyoto, Japan; Kyoto Innovation Center for Next Generation Clinical Trials and iPS Cell Therapy (Ki-CONNECT), Kyoto University Hospital, Kyoto, Japan; Department of Clinical Laboratory Medicine, Graduate School of Medicine, Kyoto University, Kyoto, Japan; Department of Life Science Frontiers, Center for iPS Cell Research and Application (CiRA), Kyoto University, Kyoto, Japan; Department of Clinical Laboratory Medicine, Graduate School of Medicine, Kyoto University, Kyoto, Japan; Department of Early Clinical Development, Graduate School of Medicine, Kyoto University, Kyoto, Japan; Kyoto Innovation Center for Next Generation Clinical Trials and iPS Cell Therapy (Ki-CONNECT), Kyoto University Hospital, Kyoto, Japan; Department of Medical Oncology, Graduate School of Medicine, Kyoto University, Kyoto, Japan; Clinical Bio-Resource Center, Kyoto University Hospital, Kyoto, Japan; Kyoto Innovation Center for Next Generation Clinical Trials and iPS Cell Therapy (Ki-CONNECT), Kyoto University Hospital, Kyoto, Japan; Department of Biomedical Statistics and Bioinformatics, Graduate School of Medicine, Kyoto University, Kyoto, Japan; Department of Life Science Frontiers, Center for iPS Cell Research and Application (CiRA), Kyoto University, Kyoto, Japan; Laboratory of Immunobiology, Graduate School of Medicine, Kyoto University, Kyoto, Japan; Kyoto University Immunomonitoring Center (KIC), Kyoto, Japan

**Keywords:** immunogenicity, multivariate linear regression analysis, prediction of vaccine response, SARS-CoV-2, vaccine efficacy

## Abstract

Despite the high effectiveness of the coronavirus disease 2019 (COVID-19) mRNA vaccines, both immunogenicity and reactogenicity show substantial interindividual variability. One key challenge is predicting high and low responders using easily measurable parameters. In this study, we performed multivariate linear regression analysis, which allows adjustment for confounding, to explore independent predictive factors for antibody responses. Using data from 216 healthy vaccinated donors aged 23–81 years, we evaluated baseline characteristics, prevaccination blood and T-cell phenotypes, and post-vaccination T-cell responses as variables, with anti-receptor-binding domain (RBD) immunoglobulin G (IgG) titers following two doses of BNT162b2 vaccination as the primary outcome. Consistent with previous reports, higher age, a history of allergic disease, and autoimmune disease were associated with lower peak IgG titers. Additionally, the frequencies of interferon-γ^+^ spike-specific CD4^+^ T cells (T-cell response) following the first vaccination strongly correlated with higher IgG responses, while those of pre-existing spike-reactive T cells showed no association with peak IgG titers. Furthermore, we identified lower percentages of naïve CD8^+^ T cells, lower hemoglobin levels, lower lymphocyte counts, and higher mean corpuscular volume as independent pre-vaccination predictors of lower peak IgG levels. Notably, the frequency of naïve CD8^+^ T cells showed a positive correlation with the peak IgG levels even in univariate analysis. These findings contribute to the individualized prediction of mRNA vaccine efficacy and may provide insights into the mechanisms underlying individual heterogeneity in immune responses.

## Teaser

Novel predictors of IgG responses to a COVID-19 mRNA vaccine

## Introduction

The newly developed coronavirus disease 2019 (COVID-19) mRNA vaccine showed significant and promising efficacy in a nationwide vaccination setting. Antibodies are the primary immune effector that confer protection against infection and promote the eradication of the severe acute respiratory syndrome coronavirus 2 (SARS-CoV-2). However, there is considerable interindividual variation in the postvaccination antibody responses (1–3). Therefore, it is necessary to identify factors that influence vaccine immunogenicity and can help predict vaccine outcomes after COVID-19 mRNA vaccination. Previous studies, which mainly focused on general medical parameters, have shown that age is among the most significant and prominent factors for predicting lower peak antibody titers (4). Other factors that are potentially negatively associated with lower post-vaccination antibody titers include male sex, use of immunosuppressive medications, excess adiposity, smoking history, hypertension, and comorbidities, such as autoimmune diseases (4–8). However, certain older adults exhibit high antibody responses, while others show the opposite. Therefore, multiple as-yet-undetermined factors could affect the individual heterogeneity of the postvaccination immune response. Recent studies have implied that immune signatures measured at prevaccination may predict the immune responses to various vaccines (9). Notably, the baseline immune-cell population frequencies, which can be measured easily, predict vaccine outcomes and protection against symptomatic infection in the context of influenza (10, 11).

T cells play a central role in antigen-specific immune responses induced by infection and vaccination. CD4^+^ helper T cells activate B cells to induce antibody production and activate phagocytic cells, whereas CD8^+^ T cells kill the virus-infected cells. The functionally distinct CD4^+^ and CD8^+^ T-cell subsets are further stratified into subpopulations of differentiation stages: naïve T cells are antigen-unexperienced cells with a diverse T-cell receptor repertoire. Thus, the naïve T-cell compartment ensures reactivity against previously unexposed or novel antigens, such as SARS-CoV-2. Memory phenotype (MP) T cells, being antigen-experienced, mediate secondary responses that facilitate rapid and robust reactions to previously encountered antigens (12). Although SARS-CoV-2 is a newly emerged virus, small proportions of SARS-CoV-2-reactive MP T cells are detected in unexposed individuals (13–15). The pre-existing so-called cross-reactive T cells, which may have been induced by exposure to the common-cold coronaviruses and/or unrelated antigens, such as microbiota (16, 17), may play a protective or pathogenic role after SARS-CoV-2 infection as well as vaccine-induced immune responses (17, 18). Despite their critical roles, the production of new T cells begins to decline during early life stages because of thymic involution and undergoes various qualitative and compositional changes and functional dysregulation with age (19–21). For example, proportions of naïve T cells decrease, and those of functionally dysregulated T cells, such as senescent, exhausted, or CD28^−^ T-cell compartments, which may be mostly generated during repeated antigen stimulation or stress, increase with age (22, 23). Notably, these T-cell phenotypes exhibit significant age-related as well as interindividual differences (15), and these factors are utilized as biomarkers of immunosenescence or immune-risk phenotypes (24). Therefore, the individual heterogeneity of pre-vaccination immune status, especially T-cell phenotypes, may influence the vaccine outcomes.

On the basis of this background, this study aimed to explore a model for identifying unknown pre-vaccination predictors of COVID-19 mRNA vaccine-induced antibody production, mainly from T-cell phenotypes. We also investigated whether complete blood counts (CBCs), commonly used in initial immune status screening, can predict antibody responses. For this purpose, we employed multiple regression analysis, a statistical approach that allows confounding adjustment by simultaneously including variables in a model (25–27). Moreover, we set up three clinical settings according to the available explanatory variables. The first was a situation in which only clinical information obtained during the prevaccine interview could be used; the second was a situation in which information obtained during the prevaccine blood draw, i.e. CBCs and T-cell phenotype information, could also be used; and the third was a situation in which the results of postvaccine immune response analysis were added. Our results may enhance the accuracy of forecasting vaccine outcomes in various clinical settings and help develop personalized vaccine strategies, including optimizing antigen doses and booster frequencies.

## Methods

### Study design and population

**Figure 1.**
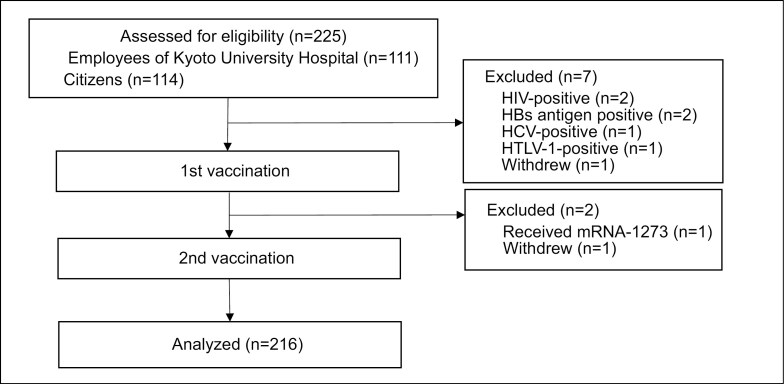
Study cohort. A total of 225 individuals were enrolled with the number of individuals meeting exclusion criteria displayed.

We analyzed data from a prospective observational cohort study (28), which enrolled 225 general citizens and employees of Kyoto University Hospital who were scheduled to receive two doses of BNT162b2 between May and August 2021. The cohort comprised adults (aged ≥20 years) without major pre-existing medical conditions. Individuals on medications such as immunomodulators and steroids, which could influence white blood cell counts and immune function, those testing positive for infectious agents [human immunodeficiency virus (HIV), hepatitis B virus, hepatitis C virus, human T-lymphotropic virus type 1 (HTLV-1)], those with test results considered inappropriate by the principal investigator or study coordinator, and recipients of the mRNA-1273 (Moderna vaccine) were excluded from this analysis (Fig. 1). We analyzed data from a cohort of 216 individuals, aged 23-81 years (28), using multivariate linear regression analysis.

### Data collection

**Figure 2.**
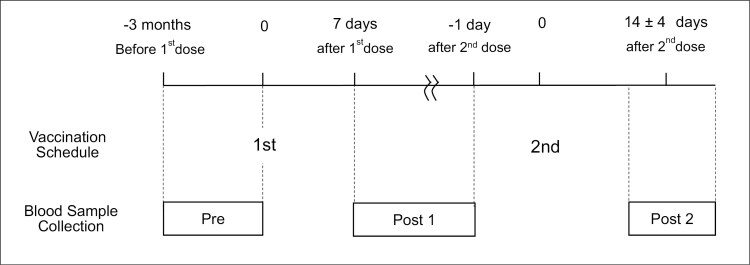
Study design. Participants received the first BNT162b2 dose on day 0 and the second on around day 21. The sampling points were set with an allowance: 7–21 days after the first dose (Post 1), 31–39 days after the first dose (Post 2). The actual vaccinated and sampling days are described in the *Results* section.

Blood samples were collected at three timepoints as follows (Fig. 2) (28): (i) Pre: up to 3 months before vaccination; (ii) Post 1: between the first and second vaccination doses, from 7 days after the first dose until the day before the second dose; and (iii) Post 2: 2 weeks after the second dose (±4 days). Baseline characteristics were collected through face-to-face physician interviews at the time of enrollment.

### Study outcome

The primary outcome was the serum anti-receptor-binding domain (RBD) immunoglobulin G (IgG) concentration after two doses of vaccination, which was highly correlated with the neutralizing activity of the original viral strain (29). Anti-SARS-CoV-2 RBD IgG levels were measured using the ARCHITECT SARS-CoV-2 IgG II Quant (Abbott, USA). The cutoff value was 50 AU/mL, based on reference values that were reported previously (28).

### Analysis of T-cell and blood samples

Data on T-cell phenotypes, such as percentages of naïve or CD28^−^ fractions in CD4^+^ or CD8^+^ T cells, the CD4/8-T-cell ratio, and percentages of vaccine antigen (spike proteins of SARS-CoV-2) -reactive or -specific CD4^+^ T cells from the previous study (28), were used as variables. The T-cell phenotypic analysis and spike-specific T-cell detection were performed as described previously (28, 30). Briefly, peripheral blood mononuclear cells (PBMCs) were stimulated for 23 h with overlapping peptides covering the complete protein-coding sequence (amino acids 5–1,273) of the spike protein (Miltenyi Biotec). These PBMCs were subsequently stained with antibodies (28) and analyzed using Northern Light 3000 and SpectroFlo version 2.2 (Cytek Biosciences). Spike-reactive or -specific T cells were defined as interferon (IFN)-γ^+^ T cells after the peptide stimulation, with background subtraction from paired distilled water controls (28). T-cell differentiation statuses were defined as naïve (Naïve: CD45RA^+^CCR7^+^CD28^+^CD95^−^) (31), central memory (CD45RA^−^CCR7^+^), effector memory (EM; CD45RA^−^CCR7^−^), or terminally differentiated EM cells re-expressing CD45RA (TEMRA; CD45RA^+^CCR7^−^). The CD28^−^ CD8^+^ T cell percentages and CD4/CD8 T cell ratio, the biomarkers of immunosenescence and immune-risk phenotypes, respectively, were also identified (22, 32). Gating strategies were indicated in Supplementary Fig 1. All flow cytometry samples were analyzed using eight separate experiments; samples from each donor obtained at all time points (Pre, Post1, Post2) were simultaneously analyzed to minimize inter-assay variation for the assays (28).

CBC was performed using an automated hematology analyzer XN-9000 (Sysmex). Anti-CMV IgG and nonspecific IgE levels were measured using chemiluminescence and fluorescence enzyme immunoassays, respectively, by LSI Medience (Tokyo, Japan). The cutoff index of anti-CMV IgG and nonspecific IgE were 1.0 AU/ml and 5.0 IU/ml, respectively.

### Statistical analysis

Summary statistics were presented as frequencies and proportions for categorical variables and as means with standard deviations or medians with interquartile ranges (IQRs) for continuous variables.

Multivariate linear regression analysis was employed, as it allows for the simultaneous examination of the effects of multiple explanatory variables on the outcome while adjusting for potential confounding factors, thereby providing more reliable estimates of independent predictors. In this approach, the degree of effect on the outcome is represented by the magnitude of the coefficient. Additionally, explanatory variables thought to contribute to confounding can be included in the model simultaneously, allowing for their independent effect on the outcome without the influence of confounding.

The analysis was conducted in three different settings. First, baseline characteristics and vaccination conditions were modeled. Next, pre-vaccination CBC test data, T-cell phenotypes, and pre-existing spike-reactive CD4^+^ T cells were incorporated. Finally, post-vaccination spike-specific T cells (T-cell responses) were added to the model. Explanatory variables included baseline characteristics [age, sex, body mass index (BMI), serology, medical history, and comorbidities], vaccine administration timing (AM or PM), pre-vaccination CBC test data [hemoglobin, mean corpuscular volume (MCV), platelet count, monocyte count, basophil count, eosinophil count, lymphocyte count, and neutrophil count], and T-cell phenotypes (naïve% in CD4^+^ or CD8^+^ T cells, CD4/8 ratio, and CD28^−^ % in CD8^+^ T cells), as well as pre-vaccination spike-reactive CD4^+^ T cells and post-vaccination spike-specific CD4^+^ T cells. These variables were selected based on prior studies suggesting their potential impact on post-vaccination antibody titers (2, 4, 7, 33–35). Since antibody levels can vary depending on the time elapsed after vaccination, the interval to blood sampling (days since the first vaccination and days since the second vaccination) was included as an explanatory variable to adjust for variations in blood sampling timing. To evaluate multicollinearity among the explanatory variables included in the model, variance inflation factor (VIF) values were calculated for each variable. A VIF value of 10 or higher is generally considered indicative of multicollinearity, while a VIF value of 5 or lower suggests a very low likelihood of multicollinearity (36).

Furthermore, a stepwise method was employed to identify the optimal model for predicting the outcome. This approach iteratively selects the best combination of predictors by adding or deleting explanatory variables from the model, aiming to find the best combination of explanatory variables for which the statistical evaluation index (the Akaike Information Criterion: AIC) is calculated to be the highest. Among the many explanatory variables considered in this study, this method was used to identify variables with greater predictive importance. In this analysis, the inclusion of a variable in the selected model carries greater interpretative significance than its p-value, as it highlights the variable’s potential as an adjusted predictor.

Supplemental analysis displayed the distribution of outcomes for variables identified as independent predictors in the multivariate regression analysis. Wilcoxon’s rank-sum tests were applied for group comparisons of categorical variables, while Pearson’s correlation coefficients were calculated for continuous variables. All statistical analyses were performed using R version 3.6.3 (R Foundation for Statistical Computing, Vienna, Austria).

### Ethics statement

This study was approved by the Ethics Committee of the Graduate School and Faculty of Medicine, Kyoto University (approval number: R0418) and was conducted in conformance with the Declaration of Helsinki and Ethical Guidelines for Medical and Health Research Involving Human Subjects. All the participants provided written informed consent to participate in the study.

## Results

### Study population

A total of 225 potential participants were screened, among whom 216 met the eligibility criteria (28, 37). The eligibility assessment is shown in Fig. 1. A summary of participant characteristics at the time of enrollment and vaccination condition is presented in Table 1. The mean age of the study participants was 56.6 years; 27.3% of the donors were over 70 years old, and 45.4% were male. Medical history was more frequent in the following order: benign diseases (27.3%); malignant tumors (11.6%); lifestyle diseases, diabetes, or vascular disorders (4.2%), and allergies, asthma, or atopic dermatitis (2.3%). Complications were more frequent in the following order: lifestyle diseases, diabetes, and vascular disorders (25.5%); allergies, asthma, and atopic dermatitis (18.5%); benign diseases (13.5%); and autoimmune disorder (3.2%), which included autoimmune disorders such as Graves’ disease, hypothyroidism, Hashimoto’s thyroiditis, IgA nephropathy, and rheumatoid arthritis. The most frequently prescribed medications were for lifestyle-related diseases, diabetes, and vascular disorders (27.8%), followed by antiallergy medications (13.4%) and other medications (25.5%), which include thyroid hormones, topical steroids, and irregular oral medications. The median number of days since vaccination was 34 and 13 for the first and second doses, respectively, with approximately 40% of participants vaccinated by noon for both doses. None of the participants had a history of COVID-19 infection, as assessed from the results of anti-SARS-CoV-2 nucleocapsid (N) protein IgM/IgG test and medical interviews.

**Table 1.** Participants' characteristics.

Characteristic	*n* = 216
Age, Mean (SD)	56.6 (15.7)
Age group, no (%)	
<35 years	25 (11.6%)
35–70 years	132 (61.1%)
≧70 years	59 (27.3%)
Male, no (%)	98 (45.4%)
BMI [kg/m^2^], Mean (SD)	22.4 (3.3)
BMI [kg/m^2^] group, no (%)	
<18.5	16 (7.4%)
18.5–25	165 (76.4%)
≧25	35 (16.2%)
Serology	
Nonspecific IgE [IU/mL], Mean (SD)	47.1 (30.4)
Cytomegalovirus-IgG antibody-positive, no (%)	173 (80.1%)
Past medical history, no (%)	
Allergies, asthma, atopic dermatitis	5 (2.3%)
Malignant tumor	25 (11.6%)
Lifestyle diseases, diabetes, vascular disorders	9 (4.2%)
Other benign diseases	59 (27.3%)
Complication, no (%)	
Allergies, asthma, atopic dermatitis	40 (18.5%)
Autoimmune disorder	7 (3.2%)
Lifestyle diseases, diabetes, vascular disorders	55 (25.5%)
Other benign diseases	33 (15.3%)
Medication, no (%)	
Allergies, asthma, atopic dermatitis	29 (13.4%)
Lifestyle diseases, diabetes, vascular disorders	60 (27.8%)
Others	55 (25.5%)
Vaccination	
Days since 1st vaccination, Median (Min, Max)	34 (30, 39)
Days since 2nd vaccination, Median (Min, Max)	13 (3, 18)
1st vaccine received in AM, no (%)	84 (39.6%)
2nd vaccine received in AM, no (%)	87 (41.0%)
With a history of COVID-19, no (%)	0 (0.0%)

Abbreviations: BMI, body mass index; COVID-19, Coronavirus disease 2019.

Cytomegalovirus-IgG antibody was regarded as positive at 6.0 or higher. The “Others” category of medication included thyroid hormones, topical steroids, and irregular oral medications.

### Primary outcome (anti-RBD IgG) and T-cell phenotypes and responses

**Figure 3.**
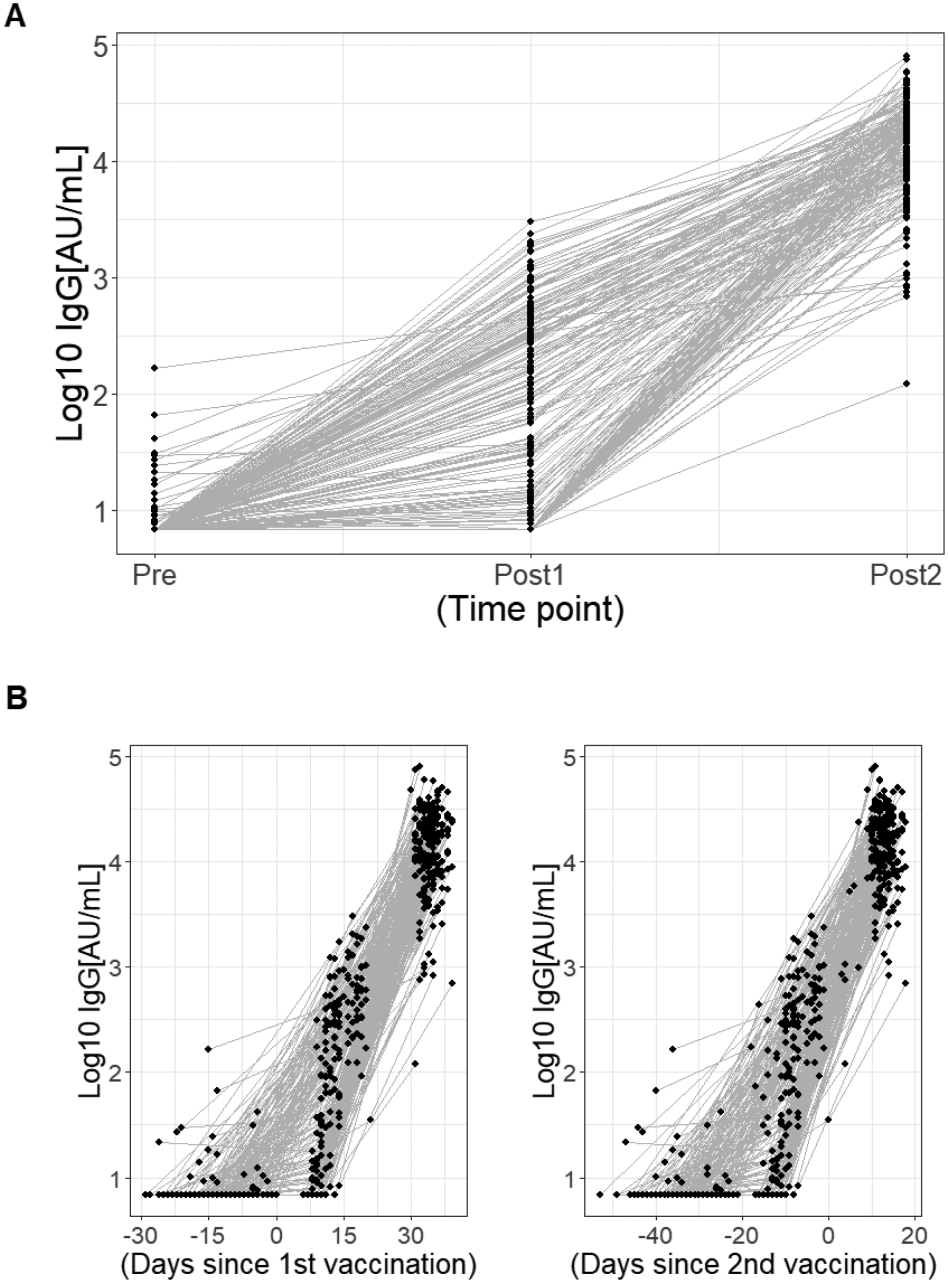
Changes in anti-RBD IgG levels. (A) Anti-RBD IgG levels at each time point (pre-vaccination, following the first dose, and following the second dose). (B) Anti-RBD IgG levels plotted by days since the first and second vaccinations, with day 0 set as the date of each respective dose.

The plot of the titers of IgG antibodies to the RBD domain of SARS-CoV-2 Spike (S1) protein at different time points after vaccination, which is the outcome of this study, is shown in Fig. 3A. The changes in log IgG by days since the first vaccination and days since the second vaccination are shown in Fig. 3B. As shown in previous studies (4, 33, 34), the IgG titers started to increase after the first vaccination and peaked after the second one.

A summary of the CBC test results, T-cell phenotypes before vaccination, and IFNγ^+^ CD4^+^ T-cell percentages before and after vaccination (spike proteins of SARS-CoV-2 (spike)-reactive or -specific CD4^+^ T cells) is shown in Table 2. The distributions of each T-cell phenotype and spike-reactive or -specific T-cell percentages at each timepoint are shown in Supplementary Figs 2 and 3, respectively. The pre-vaccination CBC was mostly distributed within the normal range, and no participant had abnormal values (Table 2). The percentage of antigen-specific T cells is generally approximately one to ten per million CD4^+^ T cells (38), and indeed, the T-cell phenotype was stable in distribution throughout the time points (Supplementary Fig. 2). In contrast, the IFNγ^+^ spike-specific T cells significantly increased after vaccination (Supplementary Fig. 3), thereby confirming that BNT162b2 vaccination induces Type 1 T helper responses (1). The percentage of IFNγ^+^ spike-specific CD4^+^ T cells was generally higher after the second dose than after the first (Supplementary Fig. 3), suggesting their proliferation and functional maturation following the second dose.

**Table 2.** Summary of complete blood tests results, T-cell phenotypes, and spike-reactive or -specific CD4^+^ T-cell percentages.

		visit	*n* = 216
Complete Blood Count, Median (IQR)	HGB [g/dl]	Pre	13.7 (12.9, 14.9)
	MCV [fl]		92.2 (90.1, 95.0)
	PLT [10^9^/l]		234.0 (202.5, 269.0)
	MONO [%]		7.0 (5.7, 8.0)
	BASO [%]		0.7 (0.5, 0.9)
	EOSINO [%]		2.1 (1.3, 3.2)
	LYMPH [10^9^/l]		1.6 (1.3, 2.0)
	NEUT [10^9^/l]		3.1 (2.6, 3.8)
T-cell phenotype, Median (IQR)	Naive in CD4 [%]	Pre	41.5 (33.3, 53.6)
	Naive in CD8 [%]		21.0 (8.3, 38.0)
	CD4/8 ratio		2.6 (1.9, 3.7)
	CD28^−^ in CD8 [%]		37.2 (25.3, 47.6)
T-cell responses, Median (IQR)	IFNg^+^ in CD4 [%]	Pre	0.01 (-0.0007, 0.02)
		Post1	0.2 (0.1, 0.5)
		Post2	0.4 (0.2, 0.6)

Abbreviations: HGB, hemoglobin; MCV, mean corpuscular volume; PLT, platelet count; MONO, monocyte; BASO, basophil count; EOSINO, eosinophil count; LYMPH, lymphocyte count; NEUT, neutrophil count.

### Predictors in the baseline characteristics

The results of the association between prevaccination baseline characteristics and vaccination condition with log IgG levels after the second vaccination (peak IgG levels) are shown in Table 3. Regarding age, a scatter plot illustrating the relationship between age and log IgG post second dose revealed that log IgG levels were generally higher in individuals aged under 35 years, marginally lower in those aged 35–70 years, and the frequency of individuals with lower IgG levels increased further in the group over 70 years. The standard deviation was 0.20 in the group aged <35 years, 0.37 in the 35–70 years group, and 0.46 in those aged >70 years. Thus, with increasing age, the variance in IgG titers among individuals becomes larger (Supplementary Fig. 4). Given these results, age was categorized into three groups: <35 years, 35–70 years, and >70 years, and included as an explanatory variable in the multivariate model. BMI was also used as an explanatory variable and categorized into three groups according to the commonly used Japanese classification of body shape: underweight (<18.5 kg/m^2^), standard weight (18.5–25 kg/m^2^), and obese (>25 kg/m^2^). Only categories with a proportion exceeding 5% of the total sample were included as explanatory variables for medical history, complications, and medications. To account for variation in blood sampling dates, the number of days post first or second vaccination was also added as an explanatory variable in all models to mitigate the potential confounding effects on the results. The VIF values of all explanatory variables were 2.5 or below, indicating the absence of multicollinearity (Table 3).

**Table 3.** Multivariate analysis of baseline characteristics and their association with anti-RBD IgG levels following the second vaccination.

Variable		Full model				Selected model		
		Regression coefficient	95% CI	*P*-value	VIF	Regression coefficient	95% CI	*P*-value
Age group	<35 years	ref	–	–	1.122	ref	–	–
	35–70 years	−0.1597	−0.3334, 0.0140	.071		−0.1558	−0.3156, 0.0040	.056
	≧70 years	−0.3871	−0.5899, −0.1844	.000		−0.4044	−0.5837, −0.2250	.000
Past medical history	Allergies/Asthma/Atopic dermatitis	−0.1771	−0.5308, 0.1767	.325	1.054			
	Malignant tumor	−0.0834	−0.2578, 0.0911	.347	1.104			
	Lifestyle diseases/Diabetes/Vascular disorders	0.0798	−0.2119, 0.3715	.590	1.154			
	Other benign diseases	−0.0187	−0.1410, 0.1035	.763	1.069			
Complication	Allergies/Asthma/Atopic dermatitis	0.0289	−0.1788, 0.2366	.784	1.593			
	Autoimmune disorder	−0.3551	−0.6824, −0.0278	.034	1.065	−0.3854	−0.6872, −0.0836	.013
	Lifestyle diseases/Diabetes/Vascular disorders	−0.1135	−0.3458, 0.1187	.336	1.971			
	Other benign diseases	−0.0021	−0.1763, 0.1720	.981	1.238			
Medication	Allergies/Asthma/Atopic dermatitis	0.0590	−0.1785, 0.2964	.625	1.600			
	Lifestyle diseases/Diabetes/Vascular disorders	0.1046	−0.1318, 0.3409	.384	2.065			
	Others	−0.0601	−0.2024, 0.0822	.406	1.215			
Sex Female		0.0073	−0.1025, 0.1171	.896	1.073			
BMI [kg/m^2^] group	<18.5	−0.0561	−0.2674, 0.1551	.601	1.073			
	18.5–25	ref	–	–				
	≧25	0.0167	−0.1344, 0.1677	.828				
Vaccination	Days since 1st vaccination	−0.0541	−0.0963, −0.0120	.012	1.585	−0.0572	−0.0970, −0.0175	.005
	Days since 2nd vaccination	0.0510	0.0180, 0.0839	.003	1.555	0.0540	0.0234, 0.0847	.001
	1st vaccine received in AM	−0.0006	−0.2469, 0.2457	.996	2.359			
	2nd vaccine received in AM	0.0073	−0.2393, 0.2539	.954	2.377			
Serology	Cytomegalovirus-IgG antibody-positive	0.0011	−0.0007, 0.0029	.225	1.062			
	Nonspecific IgE [IU/ml]	−0.0152	−0.1510, 0.1205	.825	1.070	0.0012	−0.0005, 0.0029	.151

Abbreviations: BMI, body mass index; VIF, variance inflation factor.

The stepwise multivariate model analysis identified age, complications of autoimmune disorders, days since the first vaccination, days since the second vaccination, and nonspecific IgE levels as independent predictors of peak antibody titers (Adjusted R-squared; Full model: 0.113, selected model: 0.155) (Table 3). On the basis of the positive or negative value of regression coefficients, older age, autoimmune disease complications, and days after the first vaccination were associated with a lower IgG level after the second vaccination, whereas the number of days since the second vaccination and nonspecific IgE levels were associated with higher peak IgG levels (Table 3).

### Predictors in the baseline characteristics and prevaccination blood and T-cell phenotypes

Next, the data of pre-vaccination CBC, T-cell phenotypes, and spike-reactive T cells were added as explanatory variables to the previous analysis (Table 3). Anti-RBD IgG titer before vaccination was also included as a pre-vaccination factor. The results are shown in Table 4. Because red blood cell-related parameters, hemoglobin, hematocrit, and red blood cell count (RBC), or MCV and mean corpuscular hemoglobin, were highly correlated (correlation coefficient, *r* ≥ 0.8), we anticipated potential multicollinearity effects among these parameters. Therefore, we selected two parameters, hemoglobin and MCV, as explanatory variables, as they may reflect the oxygen-carrying capacity and quality of RBCs. For the T-cell phenotype, we included the percentages of the naïve T cells, which ensure the reactivity against unexperienced antigens, as explanatory variables. Additionally, the percentage of the CD28^−^ fraction in CD8^+^ T cells, as an increase in this population is associated with immunosenescence and reduced vaccine efficacy (22). The CD4/CD8 T-cell ratio, a biomarker of immune-risk phenotypes (32, 39), and pre-vaccination IFNγ^+^ CD4^+^ T cells, indicative of pre-existing spike-reactive T cells (pre-existed cross-reactive T cells) (14), were also considered as explanatory variables. It was confirmed that the VIF values of all explanatory variables were 2.5 or lower, indicating the absence of multicollinearity (Table 4).

**Table 4.** Multivariate analysis of baseline characteristics, prevaccination blood and T-cell phenotypes, and their association with anti-RBD IgG levels following the second vaccination.

Variable		Full model				Selected model		
		Regression coefficient	95% CI	*P*-value	VIF	Regression coefficient	95% CI	*P*-value
Age group	<35 years	ref	–	–	1.248	ref	–	–
	35–70 years	−0.0471	−0.2336, 0.1395	.619		−0.0560	−0.2175, 0.1055	.495
	≧70 years	−0.1999	−0.4299, 0.0302	.088		−0.2112	−0.4081, −0.0143	.036
Past medical history	Allergies/Asthma/Atopic dermatitis	−0.3087	−0.6761, 0.0587	.099	1.123	−0.2732	−0.5939, 0.0474	.094
	Malignant tumor	−0.0568	−0.2311, 0.1174	.521	1.132			
	Lifestyle diseases/Diabetes/Vascular disorders	0.1144	−0.1798, 0.4085	.444	1.195			
	Other benign diseases	0.0058	−0.1197, 0.1313	.928	1.126			
Complication	Allergies/Asthma/Atopic dermatitis	0.0157	−0.2003, 0.2316	.886	1.701			
	Autoimmune disorder	−0.3001	−0.6290, 0.0288	.073	1.099	−0.3097	−0.5997, −0.0196	.037
	Lifestyle diseases/Diabetes/Vascular disorders	−0.0666	−0.3107, 0.1775	.591	2.128			
	Other benign diseases	0.0332	−0.1461, 0.2125	.715	1.309			
Medication	Allergies/Asthma/Atopic dermatitis	−0.0619	−0.3072, 0.1833	.619	1.697			
	Lifestyle diseases/Diabetes/Vascular disorders	0.0525	−0.1921, 0.2972	.672	2.195			
	Others	−0.0901	−0.2326, 0.0525	.214	1.251			
Sex Female		0.0054	−0.1460, 0.1567	.944	1.514			
BMI [kg/m^2^] group	<18.5	0.0356	−0.1781, 0.2493		1.144			
	18.5–25	ref	–	–				
	≧25	0.0409	−0.1194, 0.2012	.615				
Vaccination	Days since 1st vaccination	−0.0726	−0.1149, −0.0302	.001	1.634	−0.0650	−0.1028, −0.0271	.001
	Days since 2nd vaccination	0.0499	0.0172, 0.0827	.003	1.586	0.0504	0.0210, 0.0798	.001
	1st vaccine received in AM	0.0364	−0.2104, 0.2832	.771	2.428			
	2nd vaccine received in AM	−0.0109	−0.2576, 0.2357	.930	2.441			
Serology	Cytomegalovirus-IgG antibody-positive	0.0877	−0.0606, 0.2360	.245	1.190			
	Nonspecific IgE [IU/ml]	0.0004	−0.0015, 0.0022	.692	1.137			
Complete Blood Count	HGB [g/dl]	0.0232	−0.0273, 0.0737	.366	1.538	0.0253	−0.0074, 0.0580	.128
	MCV [fl]	−0.0102	−0.0223, 0.0018	.095	1.226	−0.0111	−0.0212, −0.0011	.030
	PLT [10^9^/l]	−0.0002	−0.0012, 0.0009	.746	1.228			
	MONO [%]	−0.0026	−0.0381, 0.0329	.886	1.290			
	BASO [%]	0.0202	−0.1509, 0.1913	.816	1.140			
	EOSINO [%]	0.0071	−0.0212, 0.0354	.623	1.239			
	LYMPH [10^9^/l]	0.0644	−0.0462, 0.1751	.252	1.133	0.0824	−0.0128, 0.1776	.089
	NEUT [10^9^/l]	0.0443	−0.0108, 0.0995	.114	1.240			
T-cell phenotype	Naive in CD4 [%]	0.0022	−0.0028, 0.0072	.379	1.469			
	Naive in CD8 [%]	0.0041	−0.0015, 0.0096	.147	2.122	0.0059	0.0029, 0.0090	.000
	CD4/8 ratio	−0.0202	−0.0576, 0.0171	.287	1.243			
	CD28− in CD8 [%]	−0.0022	−0.0068, 0.0024	.348	1.552			
The titers of IgG antibodies (Pre)	Log 10 IgG [AU/ml]	0.0668	−0.2756, 0.4092	.701	1.119			
Preexisting spike-reactive T cells	IFNg^+^ in CD4 [%]	0.1527	−0.3909, 0.6964	.580	1.085			

Abbreviations: HGB, hemoglobin; MCV, mean corpuscular volume; PLT, platelet count; MONO, monocyte; BASO, basophil count; EOSINO, eosinophil count; LYMPH, lymphocyte count; NEUT, neutrophil count; VIF, variance inflation factor.

Stepwise multivariate model analysis identified age, history of allergy, atopy, or asthma, autoimmune disorder complications, days since the first and second vaccinations, hemoglobin, MCV, lymphocyte count, and the percentage of naïve phenotype cells in CD8^+^ T cells as independent predictors of peak IgG titers (Adjusted R-squared; Full model: 0.163, selected model: 0.235) (Table 4). Consistent with the previous analysis (Table 3), older age, autoimmune disease complications, and the time since the first vaccination were associated with lower IgG levels following the second vaccination, whereas the time since the second vaccination was correlated with increased IgG levels. Additionally, this analysis identified a history of allergies and higher MCV levels as factors associated with lower IgG levels, while higher levels of HGB, LYMPH, and the percentage of naïve phenotype cells in CD8^+^ T cells were linked to higher IgG levels (Table 4).

### Predictors in the baseline characteristics, pre-vaccination blood and T-cell phenotypes, and post-vaccination T-cell responses

Finally, we sought to confirm whether T-cell responses, which correlate with peak IgG titers (28, 40, 41), were selected as a predictor in our stepwise multivariate model. To this end, data on spike-specific T cells after the first and second doses were added as explanatory variables to the previous analysis (Table 4). Anti-RBD IgG titers after the first dose were also included as a parameter of the postvaccination immune response. The VIF values of all explanatory variables were 2.5 or below, indicating the absence of multicollinearity (Table 5).

**Table 5.** Multivariate analysis of baseline characteristics, prevaccination blood and T-cell phenotypes, postvaccination spike-specific CD4^+^ T cells, and their association with anti-RBD IgG levels following the second vaccination.

Variable		Full model				Selected model		
		Regression coefficient	95% CI	*P*-value	VIF	Regression coefficient	95% CI	*P*-value
Age group	<35 years	ref	–	–	1.259	ref	–	–
	35–70 years	−0.0166	−0.1908, 0.1577	.851		−0.0397	−0.1899, 0.1105	.603
	≧70 years	−0.1817	−0.3948, 0.0314	.094		−0.2068	−0.3896, −0.0241	.027
Past medical history	Allergies/Asthma/Atopic dermatitis	−0.4946	−0.8414, −0.1477	.005	1.151	−0.4443	−0.7499, −0.1388	.005
	Malignant tumor	−0.0608	−0.2225, 0.1009	.459	1.140			
	Lifestyle diseases/Diabetes/Vascular disorders	0.0592	−0.2158, 0.3342	.671	1.212			
	Other benign diseases	0.0201	−0.0962, 0.1364	.734	1.133			
Complication	Allergies/Asthma/Atopic dermatitis	−0.0221	−0.2232, 0.1791	.829	1.720			
	Autoimmune disorder	−0.2623	−0.5668, 0.0422	.091	1.104	−0.2095	−0.4827, 0.0636	.132
	Lifestyle diseases/Diabetes/Vascular disorders	−0.0433	−0.2695, 0.1830	.706	2.141			
	Other benign diseases	0.0550	−0.1123, 0.2224	.517	1.326			
Medication	Allergies/Asthma/Atopic dermatitis	−0.0527	−0.2797, 0.1743	.647	1.705			
	Lifestyle diseases/Diabetes/Vascular disorders	−0.0130	−0.2403, 0.2143	.910	2.214			
	Others	−0.0866	−0.2180, 0.0448	.195	1.251	−0.0743	−0.1786, 0.0299	.161
Sex Female		−0.0763	−0.2188, 0.0661	.292	1.546	−0.0660	−0.1580, 0.0260	.158
BMI [kg/m^2^] group	<18.5	0.0731	−0.1286, 0.2749	.475	1.162			
	18.5–25	ref	–	–				
	≧25	0.0623	-0.0870, 0.2116	.411				
Vaccination	Days since 1st vaccination	−0.0638	−0.1033, −0.0244	.002	1.651	−0.0578	−0.0932, −0.0225	.001
	Days since 2nd vaccination	0.0400	0.0092, 0.0707	.011	1.616	0.0452	0.0176, 0.0727	.001
	1st vaccine received in AM	0.0775	−0.1516, 0.3067	.505	2.447			
	2nd vaccine received in AM	−0.0109	−0.2395, 0.2177	.925	2.455			
Serology	Cytomegalovirus-IgG antibody-positive	0.0069	−0.1327, 0.1464	.922	1.215			
	Nonspecific IgE [IU/ml]	0.0006	−0.0011, 0.0024	.463	1.143			
Complete Blood Count	HGB [g/dl]	0.0058	−0.0412, 0.0528	.809	1.554			
	MCV [fl]	−0.0104	−0.0216, 0.0007	.066	1.232	−0.0103	−0.0195, −0.0011	.028
	PLT [10^9^/l]	0.0001	−0.0009, 0.0011	.816	1.277			
	MONO [%]	−0.0048	−0.0376, 0.0280	.774	1.293			
	BASO [%]	0.1080	−0.0581, 0.2740	.201	1.201			
	EOSINO [%]	0.0037	−0.0226, 0.0300	.780	1.249			
	LYMPH [10^9^/l]	0.1254	0.0197, 0.2311	.020	1.175	0.1207	0.0300, 0.2115	.009
	NEUT [10^9^/l]	0.0187	−0.0329, 0.0703	.476	1.260			
T-cell phenotype	Naive in CD4 [%]	0.0004	−0.0042, 0.0051	.852	1.484			
	Naive in CD8 [%]	0.0036	−0.0015, 0.0087	.167	2.132	0.0054	0.0025, 0.0082	.000
	CD4/8 ratio	−0.0046	−0.0402, 0.0310	.799	1.287			
	CD28− in CD8 [%]	−0.0021	−0.0063, 0.0021	.328	1.553			
The titers of IgG antibodies (Pre)	Log 10 IgG [AU/ml]	−0.0308	−0.3512, 0.2896	.850	1.136			
The titers of IgG antibodies (Post 1)	Log 10 IgG [AU/ml]	0.1066	0.0460, 0.1673	.001	1.109	0.1019	0.0466, 0.1572	.000
Preexisting spike-reactive T cells	IFNg^+^ in CD4 [%]	−0.1691	−0.6839, 0.3457	.518	1.115			
T-cell response (Post 1)	IFNg^+^ in CD4 [%]	0.3154	0.1598, 0.4710	.000	1.271	0.2484	0.1193, 0.3775	.000
T-cell response (Post 2)	IFNg^+^ in CD4 [%]	0.0987	−0.0589, 0.2564	.218	1.232	0.1053	−0.0275, 0.2380	.119

Abbreviations: BMI, body mass index; HGB, hemoglobin; MCV, mean corpuscular volume; PLT, platelet count; MONO, monocyte; BASO, basophil count; EOSINO, eosinophil count; LYMPH, lymphocyte count; NEUT, neutrophil count; VIF, variance inflation factor.

The stepwise multivariate model analysis identified age, history of allergy, atopy, or asthma, presence of autoimmune disorders, other medications, sex, days since the first and second vaccinations, MCV, LYMPH, IgG titers post first vaccination, naïve% in CD8^+^ T cells, and spike-specific CD4^+^ T cells after the first and second vaccinations as independent predictors of peak IgG titers (Adjusted *R*-squared; Full model: 0.289, Selected model: 0.344) (Table 5). Consistent with the previous analysis (Table 4), older age, autoimmune disease complications, a longer interval since the first vaccination, histories of allergy, atopy, or asthma, and higher MCV were associated with a smaller increase in IgG levels following the second vaccination. Conversely, higher LYMPH levels and a greater percentage of naïve phenotype cells in CD8^+^ T cells correlated with elevated peak IgG levels.

In particular, MCV and the percentage of naïve cells in CD8^+^ T cells were considered to potentially be age-related factors. Indeed, a positive correlation was observed between MCV and age (Spearman’s correlation coefficient: *rs* = 0.301, *P* < .001), while a strong negative correlation was observed between the percentage of naïve CD8⁺ T cells and age (*rs* = −0.702, *P* < .001). Therefore, a subgroup analysis was conducted by dividing the cohort into two groups: older adults aged 65 and above and younger adults aged below 65 (Supplementary Tables 1 and 2). The results revealed that the percentage of naïve CD8⁺ T cells was identified as a predictor in the older adult group but not in the younger adult group. Conversely, MCV was identified as a predictor in the younger adult group but not in the older adult group (Supplementary Table 1: Adjusted R-squared; Full model: 0.205, Selected model: 0.377, Supplementary Table 2: Adjusted R-squared; Full model: 0.419, Selected model: 0.487).

Additionally, this analysis found that other medications were associated with a smaller increase in IgG levels. Notably, it was confirmed that higher T-cell responses after the first and second vaccinations, as well as IgG titer after the first vaccination, were strongly associated with higher IgG levels following the second dose, with *P*-values being particularly low after the first dose (*P* = .000) (Table 5).

The distributions of peak IgG levels for the categorical predictors selected in the multivariate analysis (Tables 3–5) are presented in Supplementary Fig. 5. For continuous predictors, scatterplots and correlation coefficients with peak IgG levels are shown in Supplementary Fig. 6. While most predictors showed no significant correlation with peak IgG titers (*r* ≤ 0.2), MCV exhibited a weak negative correlation (*rs* = −0.231, *P* ≤ .001). Additionally, the percentage of naïve CD8^+^ T cells (*rs* = 0.388, *P* ≤ .001) and spike-specific CD4^+^ T cells following the first vaccination (*rs* = 0.456, *P* ≤ .001) demonstrated moderate correlations (*r* ≥ 0.3) in univariate analysis.

## Discussion

Most previous studies have focused on clinical background factors as potential predictors of postvaccination antibody titers. In this study, we investigated the effects of the prevaccination CBC and T-cell phenotypes, which are easily accessible and can be used to assess immune capability and detect abnormalities in various clinical settings. Therefore, we first conducted multivariate linear regression analysis using only baseline characteristics and vaccination conditions as explanatory variables. Subsequently, prevaccination CBC and T-cell phenotype data were added to identify previously unrecognized predictive factors. Finally, T-cell responses were incorporated to evaluate whether postvaccination factors previously reported to be associated with peak antibody titers would be selected as predictors in our multivariate analysis.

Our model successfully identified several well-established factors associated with postvaccination antibody response as predictors. Notably, older age was consistently selected as a predictor across all models. This finding is consistent with previous studies, including ours, which demonstrated that peak antibody titers are negatively correlated with age, particularly in individuals aged ≥80 years (2, 6, 7, 33, 42–45). The variations in the predictive power of each variable across the three analyses are likely to be due to the addition of new variables, which eliminated confounding effects. For instance, in the case of age, including T-cell phenotypes and CBC variables in the second and third settings (Tables 4 and 5) mitigated the confounding influence of these variables on age. Additionally, autoimmune complications were selected in all models. This result aligns with earlier reports that vaccination efficacy is lower in patients with autoimmune diseases, likely because of immunomodulatory treatments such as glucocorticoids, tumor necrosis factor-α inhibitors, and abatacept therapy (46). Although participants taking oral steroids or immunosuppressive drugs were excluded from this study, past use of these medications may have affected the results. Furthermore, accelerated T-cell aging in autoimmune disease patients could also contribute to this phenomenon (47). Overall, these results highlight the robustness and feasibility of our model.

On the other hand, some of the predictors identified in this study require further detailed investigation. First, although several previous reports showed that postvaccination antibody titers were higher in females than in males (7, 43), female sex was selected as a predictor of lower peak IgG titers in the model, which includes post-vaccination T cell-related variables. This discrepancy is likely due to the large individual differences observed in females, where some individuals with particularly low antibody titers were included in the group despite females generally exhibiting higher IgG levels than males (Supplementary Fig. 5). Allergy-related factors were also selected as predictors, consistent with previous reports (7). However, nonspecific IgE, typically elevated in allergic individuals, was positively associated with the outcome in the first model, whereas a medical history of allergies was negatively associated in all models. A previous report identified the medication for allergy as a predictor of higher antibody titers (7), whereas in our study, the regression coefficient for comorbidities of asthma and atopic dermatitis was negative. Additionally, the percentage of eosinophils, often elevated in allergic conditions, was not selected as a predictor in any of our models. These seemingly conflicting results suggest that allergy-related factors (e.g. history, comorbidities, medications, or allergy type) may influence antibody responses in separate and complex ways. Further investigations with a more detailed analysis of allergy-related factors are warranted. In the final model, other medications unrelated to allergies or lifestyle were also selected as predictors. This category excluded immunosuppressants but included thyroid hormones, topical steroids, and irregular oral medications. Further research is needed to clarify which drug might affect IgG responses.

As part of the vaccine conditions, we included the vaccine administration timing (AM or PM) as an explanatory variable. Previous studies reported that vaccination in the morning was more effective (48, 49), probably due to the circadian rhythm’s regulation of adaptive immune responses (50–52). However, in this study, vaccination timing was not identified as a predictor of peak IgG titers. These results may be attributed to several factors. First, all participants received their vaccines in the morning or afternoon, with none vaccinated in the evening. Secondly, the robust primary response induced by the novel mRNA vaccine may have overshadowed any differences in immune response efficiency related to vaccination timing. Indeed, the advantage of daytime COVID-19 mRNA vaccination was noted only in terms of hospitalization rates, not breakthrough infections, suggesting minimal variance in immune responses among healthy individuals (48). All models selected the number of days since the first or second vaccination, included as an explanatory variable for adjustment, as negative and positive predictors, respectively. The positive association between days since the second vaccination and antibody titers suggests that the antibody levels did not yet reach a plateau at the time of blood collection. These variables likely accounted for variations in antibody titers caused by differences in the number of days post-vaccination within the permissible range in our models.

Importantly, in our model, two RBC-related factors were newly identified as predictors: higher hemoglobin and higher MCV, which were associated with higher and lower antibody titers, respectively. Efficient oxygen delivery may facilitate efficient induction of immune responses, as lymphocytes undergo substantial proliferation, particularly in response to a sudden and strong antigenic stimulus such as a vaccine. Although a high MCV is commonly associated with insufficient vitamin B12 or folic acid, which could lead to macrocytic anemia (53), all donors in this study had MCV values within the normal range (<100 fL). Further studies are needed to investigate whether individuals with a tendency of higher MCV within the normal range tend to show lower vitamin B12 or Folate levels, which could impair DNA synthesis and integrity, subsequently reducing lymphocyte proliferation.

For T-cell phenotypes, we included well-recognized parameters that significantly change with age and/or are associated with lower vaccine responses or immune-risk phenotypes (15, 20, 21, 28, 54, 55). Among these, only the percentage of naïve CD8^+^ T cells was identified as a predictor positively associated with an increased antibody titer. This result was unexpected, as the antibody response is typically driven by CD4^+^ helper T cells rather than CD8^+^ cytotoxic T cells. One possible reason for this finding is that the age-related decline in naïve T cells is more pronounced in CD8^+^ T cells than in CD4^+^ T cells (15, 56). An age-subgroup analysis suggested that the naïve-cell % in CD8^+^ T cells is a stronger biomarker of increase of IgG titer in older adults. This is probably because the naïve-cell % in CD8^+^ T cells is maintained at relatively high levels in the group under 65 years of age. In any case, the result strongly suggests that the percentage of naïve CD8^+^ T cells is a more sensitive immunosenescence biomarker than previously recognized.

The impact of preexisting spike-reactive T-cell immunity on COVID-19 mRNA vaccine responses is controversial (17). In our model, the frequency of preexisting spike-reactive T-cells was not identified as a predictor of higher peak IgG titers. This suggests their limited impact, although the quality of pre-existing spike-reactive T cells may still influence vaccine responses. In contrast, in the model incorporating post-vaccination CD4^+^ T-cell responses as additional variables, the frequencies of spike-specific CD4^+^ T cells following the first and second doses were identified as predictors, with a stronger association observed for the first dose. This finding aligns with previous studies, including ours, which demonstrated that a rapid CD4^+^ T cell response is associated with higher humoral immunity to COVID-19 mRNA vaccination and that weaker CD4⁺ T cell responses after the first dose are observed in older adults (28, 40, 41). These results further confirm the feasibility and robustness of the current model.

This study had several limitations. First, the sample size was relatively small, and participants were recruited from a limited regional area. Future studies should include a more comprehensive range of regions and age groups to improve generalizability. Second, the medical history and comorbidities were based on participant interviews and may not represent entirely accurate information. Third, this study focused on baseline characteristics, complete blood tests, and T-cell phenotypes as candidate factors for a postvaccination increase in IgG levels. Other factors, particularly those related to B cells and antigen-presenting cells that directly involve antibody responses, were not included. Finally, we employed the stepwise method to identify predictors. While this approach is useful, it is well-documented that stepwise methods can lead to overfitting, reduce reproducibility, and potential bias in parameter estimation (57, 58). Further studies are required to evaluate the robustness and generalizability of our findings.

In conclusion, using multivariate linear regression analysis with a stepwise variable-selection method, we identified several key unrecognized predictors of inadequate antibody responses after COVID-19 mRNA vaccination. Specifically, a lower percentage of naïve CD8^+^ T cells, lower hemoglobin, lower lymphocyte counts, and higher MCV were associated with reduced antibody responses following two doses of COVID-19 mRNA vaccination. Our findings contribute to the individualized prediction of vaccine efficacy and the development of tailored vaccine doses or schedule based on individual immune status. Furthermore, this study offers potential insights into the mechanisms underlying the individual heterogeneity of immune responses.

## Supplementary Material

dxaf013_suppl_Supplementary_Figures_1-6

dxaf013_suppl_Supplementary_Tables_1-2

## Data Availability

The data underlying this article will be shared on reasonable request to the corresponding author.
