# Supplementary material for: Effect of prevaccination blood and T-cell phenotypes on antibody responses to a COVID-19 mRNA vaccine
Source: Int Immunol. 2025 Mar 21;37(7):403–16. doi: 10.1093/intimm/dxaf013 (PMC12190804; doi:10.1093/intimm/dxaf013)
Supplement: dxaf013_suppl_Supplementary_Figures_1-6 [file dxaf013_suppl_supplementary_figures_1-6.docx]

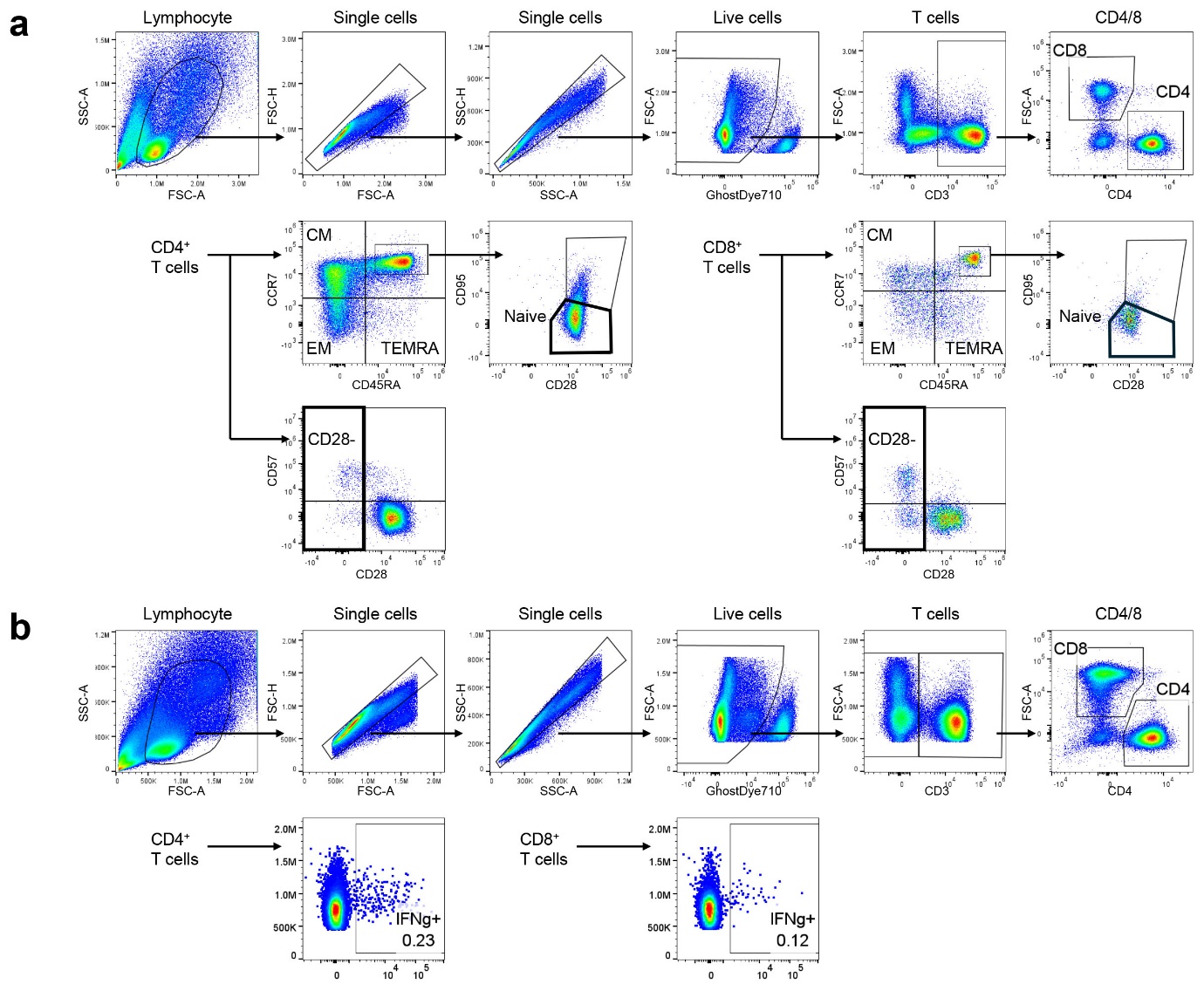


**Suppl Figure 1. Representative gating strategy for flow cytometric analysis of T-cell phenotypes.**
(a) Lymphocytes were gated, and doublets were excluded. Live cells were gated as Ghost Dye™ Red 710⁻ cells. T cells were gated as CD3⁺ and further subdivided into CD4⁺ and CD8⁺ populations. Differentiation statuses were classified as naive (Naive, CD45RA⁺CCR7⁺CD28⁺CD95⁻), central memory (CM, CD45RA⁻CCR7⁺), effector memory (EM, CD45RA⁻CCR7⁻), or terminally differentiated effector memory cells re-expressing CD45RA (TEMRA, CD45RA⁺CCR7⁻). CD28⁻ cells were also identified. (b) Representative gating strategy for detecting interferon-γ⁺ spike-reactive or spike-specific T cells.


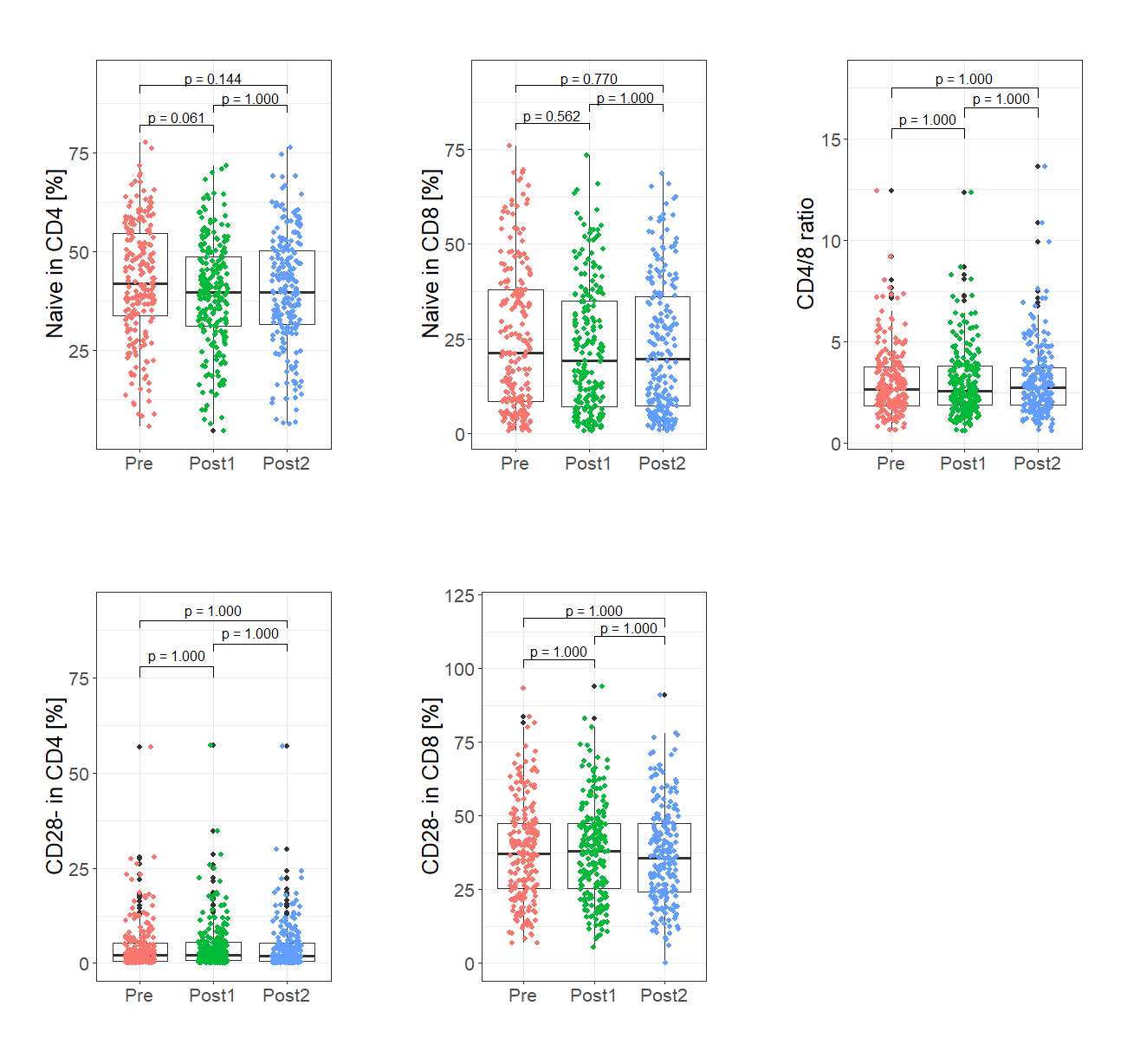


**Suppl Figure 2. Distribution of T-cell phenotypes across time points.**T-cell phenotypes were analyzed at each time point, including the percentages of naïve cells in CD4⁺ and CD8⁺ T cells, the CD4/CD8 ratio, and the percentages of CD28⁻ cells in CD4⁺ and CD8⁺ T cells.

　　　　　 　
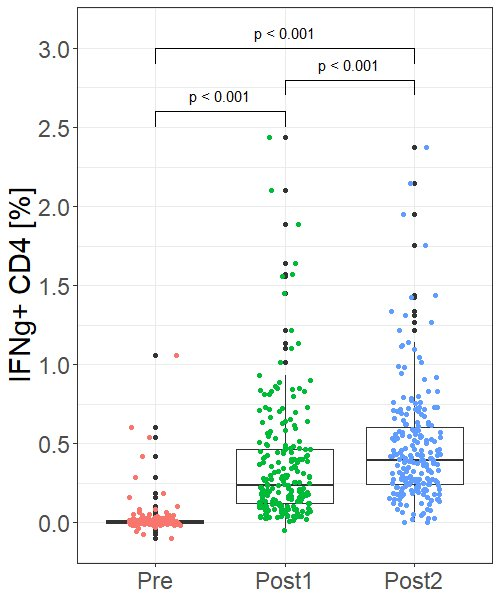


IFNγ^+^ in CD4 [%]

**Suppl Figure 3. Distribution of spike-reactive or spike-specific CD4⁺ T-cell percentages across time points.**
T-cell phenotypes were analyzed at each time point, showing the percentages of interferon-γ⁺ cells in total CD4⁺ T cells.

　
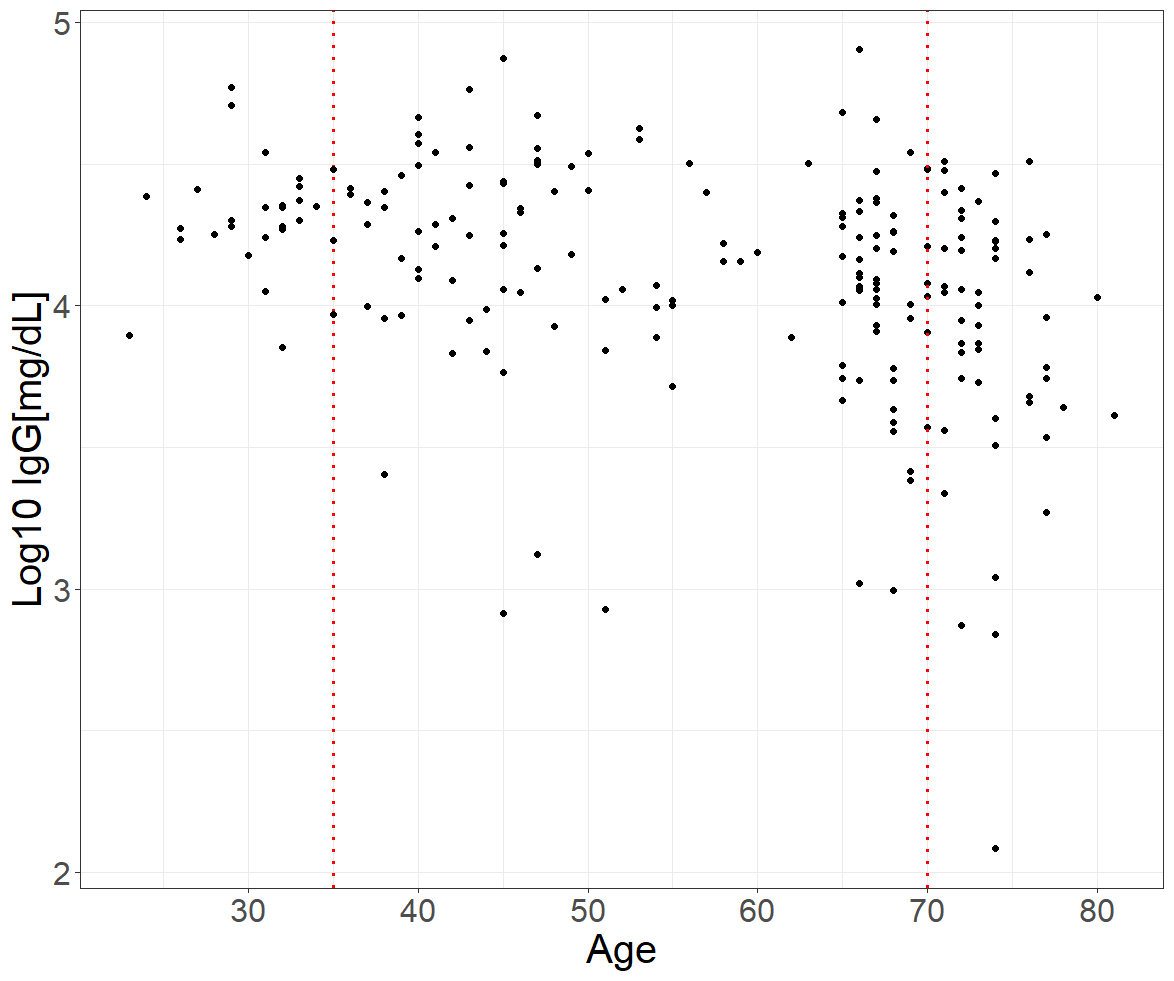


Log10 IgG [AU/mL]

Age

**Suppl Figure 4. Scatterplot of age and log-transformed IgG levels following the second vaccine dose.**
A scatterplot showing the relationship between age and log-transformed anti-RBD IgG levels following the second dose of the vaccine. The red dotted lines indicate ages 35 and 70.

Past medical history:
Allergies/Asthma/Atopic dermatitis

Age group

Sex


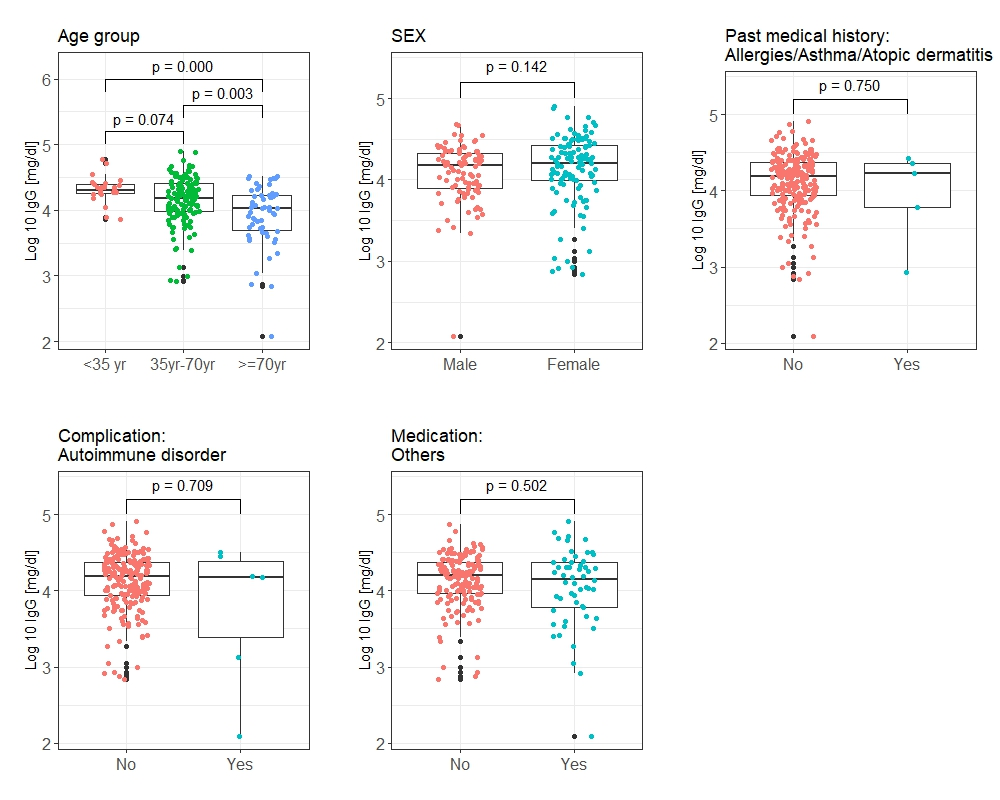


Log10 IgG [AU/mL]

Log10 IgG [AU/mL]

Log10 IgG [AU/mL]

Log10 IgG [AU/mL]

Log10 IgG [AU/mL]

<35yr 35-70yr >=70y

Male Female

No Yes

Medication:
Others

Complication:
Autoimmune disorder


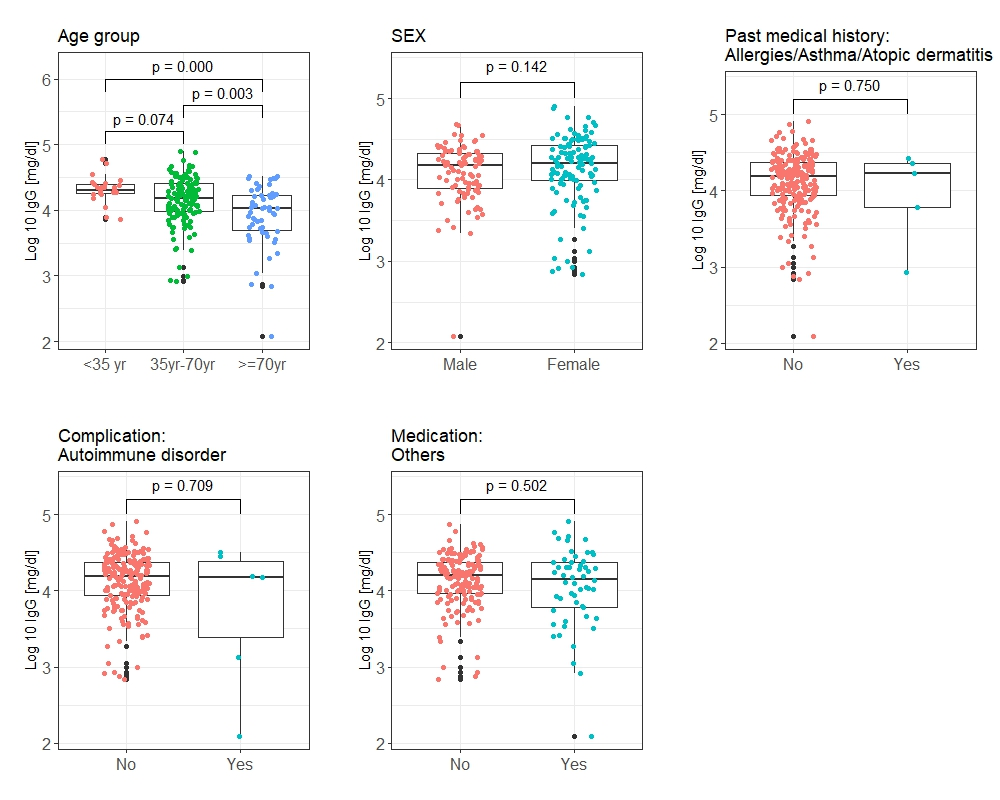


No Yes

No Yes

**Suppl Figure 5. Distribution of log-transformed IgG levels by** **categorical variables identified in the selected model.**The distribution of log-transformed IgG levels is shown for categorical variables identified as predictors in the selected model. Comparisons between variable levels were performed using Wilcoxon's rank-sum test.


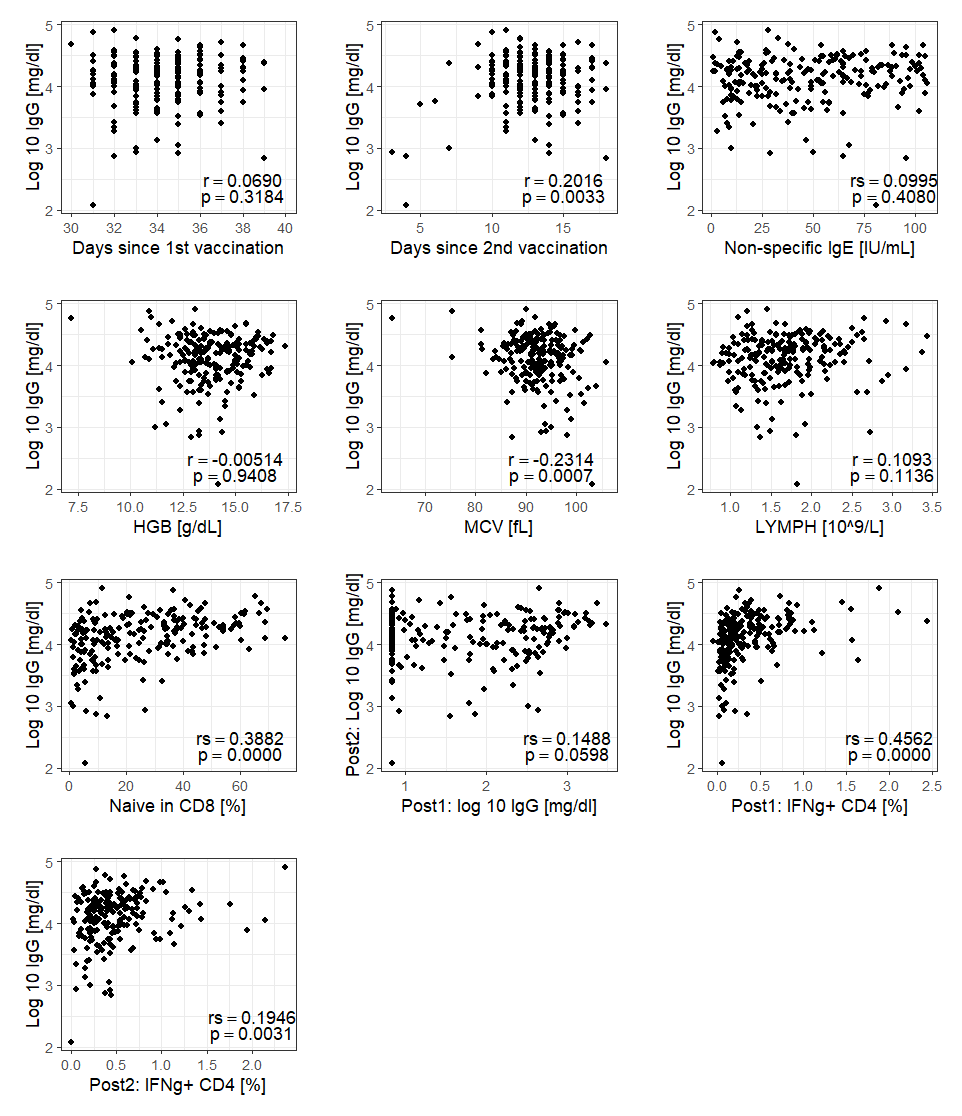


**Suppl Figure 6. Scatter plots of log -transformed IgG and continuous variables identified in the selected model.**

Scatterplots showing the relationships between log-transformed IgG levels and continuous variables identified as predictors in the selected model. Pearson’s correlation (r) or Spearman’s rank correlation (rs) were used to evaluate the relationships between variables.
