# Supplementary material for: Effect of prevaccination blood and T-cell phenotypes on antibody responses to a COVID-19 mRNA vaccine
Source: Int Immunol. 2025 Mar 21;37(7):403–16. doi: 10.1093/intimm/dxaf013 (PMC12190804; doi:10.1093/intimm/dxaf013)
Supplement: dxaf013_suppl_Supplementary_Tables_1-2 [file dxaf013_suppl_supplementary_tables_1-2.docx]

Suppl Table 1. Multivariate analysis of baseline characteristics, pre-vaccination blood and T-cell phenotypes, post-vaccination spike-specific CD4+ T cells, and their association with anti-RBD IgG levels following the second vaccination (Adult group under 65 years old).

| Variable | | Full model | | | |  | Selected model | | |
| --- | --- | --- | --- | --- | --- | --- | --- | --- | --- |
|  |  | Regression coefficient | 95%CI | p-value | VIF |  | Regression coefficient | 95%CI | p-value |
| Age group | < 35 yr | ref | - | - | 1.396 |  | ref | - | - |
|  | 35 yr - 65 yr | -0.0883 | -0.2817, 0.1050 | 0.365 |  |  | -0.1087 | -0.2494, 0.0321 | 0.129 |
| Past medical history | Allergies/Asthma/ Atopic dermatitis | -0.1093 | -0.5517, 0.3331 | 0.623 | 1.439 |  |  |  |  |
|  | Malignant tumor | 0.0545 | -0.2096, 0.3185 | 0.682 | 1.256 |  |  |  |  |
|  | Lifestyle diseases/ Diabetes/Vascular disorders | -0.1402 | -0.6243, 0.3439 | 0.565 | 1.371 |  |  |  |  |
|  | Other benign diseases | 0.0230 | -0.1861, 0.2321 | 0.827 | 1.337 |  |  |  |  |
| Complication | Allergies/Asthma/ Atopic dermatitis | -0.3146 | -0.6057, -0.0235 | 0.035 | 2.129 |  | -0.2781 | -0.4935, -0.0627 | 0.012 |
|  | Autoimmune disorder | -0.1859 | -0.6150, 0.2432 | 0.390 | 1.396 |  | -0.2363 | -0.5461, 0.0735 | 0.133 |
|  | Lifestyle diseases/Diabetes/Vascular disorders | -0.2870 | -0.8776, 0.3035 | 0.335 | 2.502 |  |  |  |  |
|  | Other benign diseases | -0.0125 | -0.2923, 0.2674 | 0.929 | 1.455 |  |  |  |  |
| Medication | Allergies/ Asthma/Atopic dermatitis | 0.3403 | 0.0663, 0.6144 | 0.016 | 1.859 |  | 0.3277 | 0.1025, 0.5530 | 0.005 |
|  | Lifestyle diseases/Diabetes/Vascular disorders | 0.1935 | -0.3484, 0.7354 | 0.478 | 2.701 |  |  |  |  |
|  | Others | -0.1141 | -0.3186, 0.0905 | 0.269 | 1.362 |  |  |  |  |
| Sex Female | | -0.2291 | -0.4917, 0.0334 | 0.086 | 2.155 |  | -0.2892 | -0.4772, -0.1012 | 0.003 |
| BMI [kg/m^2^] group | < 18.5 | -0.0567 | -0.3498, 0.2365 | 0.701 | 1.506 |  |  |  |  |
|  | 18.5 – 25 | ref | - | - |  |  |  |  |  |
|  | ≧25 | 0.0053 | -0.2747, 0.2854 | 0.970 |  |  |  |  |  |
| Vaccination | Days since 1st vaccination | -0.0355 | -0.0883, 0.0173 | 0.184 | 1.559 |  | -0.0411 | -0.0850, 0.0029 | 0.067 |
|  | Days since 2nd vaccination | 0.0117 | -0.0272, 0.0506 | 0.551 | 1.598 |  | 0.0237 | -0.0077, 0.0551 | 0.137 |
|  | 1st vaccine received in AM | 0.4430 | 0.0818, 0.8042 | 0.017 | 2.977 |  | 0.4589 | 0.1760, 0.7417 | 0.002 |
|  | 2nd vaccine received in AM | -0.2203 | -0.5659, 0.1252 | 0.207 | 2.859 |  | -0.2364 | -0.5081, 0.0352 | 0.087 |
| Serology | Cytomegalovirus -IgG antibody-positive | -0.0869 | -0.2566, 0.0827 | 0.310 | 1.344 |  | -0.0783 | -0.1989, 0.0423 | 0.200 |
|  | Non-specific IgE [IU/mL] | 0.0027 | 0.0000, 0.0054 | 0.049 | 1.393 |  | 0.0031 | 0.0012, 0.0050 | 0.002 |
| Complete Blood Count | HGB [g/dL] | -0.0519 | -0.1260, 0.0222 | 0.167 | 2.161 |  | -0.0603 | -0.1119, -0.0087 | 0.023 |
|  | MCV [fL] | -0.0026 | -0.0185, 0.0133 | 0.748 | 1.392 |  |  |  |  |
|  | PLT [10^9^/L] | 0.0016 | -0.0002, 0.0033 | 0.074 | 1.574 |  | 0.0017 | 0.0005, 0.0028 | 0.007 |
|  | MONO [%] | -0.0283 | -0.0848, 0.0281 | 0.319 | 1.430 |  | -0.0464 | -0.0858, -0.0070 | 0.021 |
|  | BASO [%] | 0.1060 | -0.1709, 0.3828 | 0.447 | 1.564 |  |  |  |  |
|  | EOSINO [%] | 0.0032 | -0.0300, 0.0365 | 0.847 | 1.38 |  |  |  |  |
|  | LYMPH [10^9^/L] | 0.0235 | -0.1444, 0.1913 | 0.781 | 1.337 |  |  |  |  |
|  | NEUT [10^9^/L] | 0.0400 | -0.0344, 0.1143 | 0.287 | 1.533 |  |  |  |  |
| T-cell phenotype | Naive in CD4 [%] | -0.0018 | -0.0091, 0.0054 | 0.620 | 1.595 |  |  |  |  |
|  | Naive in CD8 [%] | 0.0029 | -0.0044, 0.0101 | 0.433 | 1.999 |  |  |  |  |
|  | CD4/8 ratio | -0.0215 | -0.1034, 0.0605 | 0.603 | 1.443 |  |  |  |  |
|  | CD28- in CD8 [%] | 0.0033 | -0.0039, 0.0104 | 0.366 | 1.692 |  |  |  |  |
| The titers of IgG antibodies (Pre) | Log 10 IgG [AU/mL] | 0.2991 | -0.1879, 0.7861 | 0.224 | 1.331 |  | 0.3479 | -0.0354, 0.7312 | 0.075 |
| The titers of IgG antibodies (Post1) | Log 10 IgG [AU/mL] | 0.0596 | -0.0333, 0.1524 | 0.205 | 1.298 |  | 0.0594 | -0.0132, 0.1320 | 0.107 |
| Pre-existing spike-reactive T cells | IFNg^+^ in CD4 [%] | 0.0388 | -0.5628, 0.6404 | 0.898 | 1.226 |  |  |  |  |
| T-cell response (Post1) | IFNg^+^ in CD4 [%] | 0.2293 | -0.0045, 0.4630 | 0.054 | 1.414 |  | 0.2570 | 0.0787, 0.4353 | 0.005 |
| T-cell response (Post2) | IFNg^+^ in CD4 [%] | 0.1061 | -0.1461, 0.3583 | 0.404 | 1.374 |  | 0.1526 | -0.0371, 0.3422 | 0.113 |

HGB: Hemoglobin, MCH: Mean corpuscular hemoglobin, PLT: Platelet count, MONO: Monocyte, BASO: Basophil count, EOSINO: Eosinophil count, LYMPH: Lymphocyte count, NEUT: Neutrophil count, VIF: Variance inflation factor

Suppl Table 2. Multivariate analysis of baseline characteristics, pre-vaccination blood and T-cell phenotypes, post-vaccination spike-specific CD4+ T cells, and their association with anti-RBD IgG levels following the second vaccination (Elderly group over 65 years old).

| Variable | | Full model | | | |  | Selected model | | |
| --- | --- | --- | --- | --- | --- | --- | --- | --- | --- |
|  |  | Regression coefficient | 95%CI | p-value | VIF |  | Regression coefficient | 95%CI | p-value |
| Age group | 65 yr - 70 yr | ref | - | - | 1.260 |  | ref | - | - |
|  | ≧70 yr | -0.0741 | -0.2300, 0.0819 | 0.347 |  |  |  |  |  |
| Past medical history | Allergies/Asthma/ Atopic dermatitis | -0.5834 | -1.287, 0.1206 | 0.103 | 1.092 |  | -0.4674 | -1.096, 0.1614 | 0.143 |
|  | Malignant tumor | -0.1642 | -0.3912, 0.0627 | 0.153 | 1.306 |  | -0.1950 | -0.3714, -0.0187 | 0.031 |
|  | Lifestyle diseases/ Diabetes/Vascular disorders | 0.2161 | -0.1498, 0.5819 | 0.243 | 1.358 |  |  |  |  |
|  | Other benign diseases | 0.0647 | -0.1074, 0.2368 | 0.456 | 1.346 |  |  |  |  |
| Complication | Allergies/Asthma/ Atopic dermatitis | 0.3304 | 0.0025, 0.6583 | 0.048 | 1.784 |  | 0.3354 | 0.0635, 0.6074 | 0.016 |
|  | Autoimmune disorder | -0.4233 | -0.9873, 0.1407 | 0.139 | 1.232 |  | -0.4683 | -0.9341, -0.0025 | 0.049 |
|  | Lifestyle diseases/Diabetes/Vascular disorders | 0.0648 | -0.2122, 0.3419 | 0.642 | 2.219 |  |  |  |  |
|  | Other benign diseases | 0.0831 | -0.1491, 0.3153 | 0.478 | 1.515 |  |  |  |  |
| Medication | Allergies/ Asthma/Atopic dermatitis | -0.5127 | -0.9321, -0.0933 | 0.017 | 1.779 |  | -0.3986 | -0.7491, -0.0482 | 0.026 |
|  | Lifestyle diseases/Diabetes/Vascular disorders | -0.0784 | -0.3591, 0.2023 | 0.579 | 2.259 |  |  |  |  |
|  | Others | -0.0578 | -0.2599, 0.1443 | 0.570 | 1.520 |  |  |  |  |
| Sex Female | | -0.0705 | -0.2594, 0.1184 | 0.459 | 1.530 |  | -0.1121 | -0.2344, 0.0102 | 0.072 |
| BMI [kg/m^2^] group | < 18.5 | 0.1793 | -0.1280, 0.4866 | 0.249 | 1.275 |  |  |  |  |
|  | 18.5 – 25 | ref | - | - |  |  |  |  |  |
|  | ≧25 | 0.0619 | -0.1595, 0.2833 | 0.579 |  |  |  |  |  |
| Vaccination | Days since 1st vaccination | -0.1366 | -0.2046, -0.0685 | 0.000 | 2.015 |  | -0.1360 | -0.1938, -0.0782 | 0.000 |
|  | Days since 2nd vaccination | 0.1005 | 0.0456, 0.1553 | 0.001 | 2.032 |  | 0.1064 | 0.0591, 0.1536 | 0.000 |
|  | 1st vaccine received in AM | -0.1089 | -0.4301, 0.2123 | 0.501 | 2.547 |  | -0.1244 | -0.2500, 0.0012 | 0.052 |
|  | 2nd vaccine received in AM | 0.0221 | -0.2926, 0.3367 | 0.889 | 2.513 |  |  |  |  |
| Serology | Cytomegalovirus -IgG antibody-positive | 0.0020 | -0.2859, 0.2899 | 0.989 | 1.221 |  |  |  |  |
|  | Non-specific IgE [IU/mL] | -0.0020 | -0.0044, 0.0004 | 0.104 | 1.162 |  | -0.0019 | -0.0040, 0.0002 | 0.074 |
| Complete Blood Count | HGB [g/dL] | 0.0114 | -0.0694, 0.0923 | 0.779 | 1.658 |  |  |  |  |
|  | MCV [fL] | -0.0027 | -0.0195, 0.0140 | 0.745 | 1.245 |  |  |  |  |
|  | PLT [10^9^/L] | -0.0010 | -0.0026, 0.0006 | 0.232 | 1.501 |  |  |  |  |
|  | MONO [%] | -0.0066 | -0.0521, 0.0389 | 0.774 | 1.331 |  |  |  |  |
|  | BASO [%] | 0.1331 | -0.1057, 0.3719 | 0.270 | 1.268 |  | 0.1600 | -0.0414, 0.3614 | 0.118 |
|  | EOSINO [%] | 0.0243 | -0.0218, 0.0704 | 0.297 | 1.391 |  |  |  |  |
|  | LYMPH [10^9^/L] | 0.1391 | -0.0092, 0.2874 | 0.066 | 1.300 |  | 0.1166 | -0.0022, 0.2353 | 0.054 |
|  | NEUT [10^9^/L] | 0.0430 | -0.0409, 0.1269 | 0.310 | 1.337 |  |  |  |  |
| T-cell phenotype | Naive in CD4 [%] | -0.0002 | -0.0068, 0.0065 | 0.959 | 1.617 |  |  |  |  |
|  | Naive in CD8 [%] | 0.0054 | -0.0033, 0.0142 | 0.218 | 1.671 |  | 0.0067 | 0.0013, 0.0120 | 0.016 |
|  | CD4/8 ratio | -0.0050 | -0.0500, 0.0400 | 0.825 | 1.432 |  |  |  |  |
|  | CD28- in CD8 [%] | -0.0027 | -0.0084, 0.0031 | 0.360 | 1.540 |  |  |  |  |
| The titers of IgG antibodies (Pre) | Log 10 IgG [AU/mL] | -0.5387 | -1.041, -0.0366 | 0.036 | 1.320 |  | -0.6545 | -1.062, -0.2473 | 0.002 |
| The titers of IgG antibodies (Post1) | Log 10 IgG [AU/mL] | 0.1628 | 0.0633, 0.2624 | 0.002 | 1.308 |  | 0.1489 | 0.0738, 0.2239 | 0.000 |
| Pre-existing spike-reactive T cells | IFNg^+^ in CD4 [%] | -0.6532 | -1.972, 0.6660 | 0.327 | 1.549 |  | -0.9628 | -1.918, -0.0073 | 0.048 |
| T-cell response (Post1) | IFNg^+^ in CD4 [%] | 0.4456 | 0.2286, 0.6626 | 0.000 | 1.321 |  | 0.4078 | 0.2232, 0.5924 | 0.000 |
| T-cell response (Post2) | IFNg^+^ in CD4 [%] | 0.1963 | -0.0729, 0.4655 | 0.150 | 1.693 |  | 0.2737 | 0.0796, 0.4678 | 0.006 |

HGB: Hemoglobin, MCH: Mean corpuscular hemoglobin, PLT: Platelet count, MONO: Monocyte, BASO: Basophil count, EOSINO: Eosinophil count, LYMPH: Lymphocyte count, NEUT: Neutrophil count, VIF: Variance inflation factor
